# Rural-Urban Inequity in Unmet Obstetric Needs and Functionality of Emergency Obstetric Care Services in a Zambian District

**DOI:** 10.1371/journal.pone.0145196

**Published:** 2016-01-29

**Authors:** Selia Ng’anjo Phiri, Knut Fylkesnes, Karen Marie Moland, Jens Byskov, Torvid Kiserud

**Affiliations:** 1 Centre for International Health, Department of Global Public Health and Primary Care, University of Bergen, Bergen, Norway; 2 Department of Public Health, School of Medicine, University of Zambia, Lusaka, Zambia; 3 Research Unit for Human Parasitology and the Environment, Faculty of Health and Medical Sciences, University of Copenhagen, Dyrlaegevej 100, DK-1870 Frederiksberg C, Copenhagen, Denmark; 4 Department of Obstetrics and Gynaecology, Haukeland University Hospital, Bergen, Norway; 5 Department of Clinical Science, University of Bergen, Bergen, Norway; Iran University of Medical Sciences, ISLAMIC REPUBLIC OF IRAN

## Abstract

**Background:**

Zambia has a high maternal mortality ratio, 398/100,000 live births. Few pregnant women access emergency obstetric care services to handle complications at childbirth. We aimed to assess the deficit in life-saving obstetric services in the rural and urban areas of Kapiri Mposhi district.

**Method:**

A cross-sectional survey was conducted in 2011 as part of the ‘Response to Accountable priority setting for Trust in health systems’ (REACT) project. Data on all childbirths that occurred in emergency obstetric care facilities in 2010 were obtained retrospectively. Sources of information included registers from maternity ward admission, delivery and operation theatre, and case records. Data included age, parity, mode of delivery, obstetric complications, and outcome of mother and the newborn. An approach using estimated major obstetric interventions expected but not done in health facilities was used to assess deficit of life-saving interventions in urban and rural areas.

**Results:**

A total of 2114 urban and 1226 rural childbirths occurring in emergency obstetric care facilities (excluding abortions) were analysed. Facility childbirth constituted 81% of expected births in urban and 16% in rural areas. Based on the reference estimate that 1.4% of childbearing women were expected to need major obstetric intervention, unmet obstetric need was 77 of 106 women, thus 73% (95% CI 71–75%) in rural areas whereas urban areas had no deficit. Major obstetric interventions for absolute maternal indications were higher in urban 2.1% (95% CI 1.60–2.71%) than in rural areas 0.4% (95% CI 0.27–0.55%), with an urban to rural rate ratio of 5.5 (95% CI 3.55–8.76).

**Conclusions:**

Women in rural areas had deficient obstetric care. The likelihood of under-going a life-saving intervention was 5.5 times higher for women in urban than rural areas. Targeting rural women with life-saving services could substantially reduce this inequity and preventable deaths.

## Introduction

Access to emergency obstetric and neonatal care (EmONC) and skilled birth attendance are important elements of strategies to reducing maternal and neonatal mortality [1,2,3]. The fifth WHO Millennium Development Goal (MDG5) aimed to reduce maternal mortality by 75% by 2015 but made the least progress among the MDGs, that is 45% was achieved from 1990 levels of 543,000 maternal deaths [4]. Globally, 289,000 women died from pregnancy related complications in 2013 [4], half of them due to haemorrhage, hypertensive disorders and sepsis [5]. The majority occurred just before, during or after birth [6], signifying that the need for skilled birth attendance and access to emergency obstetric and neonatal care had not been met by far.

**Fig 1:**
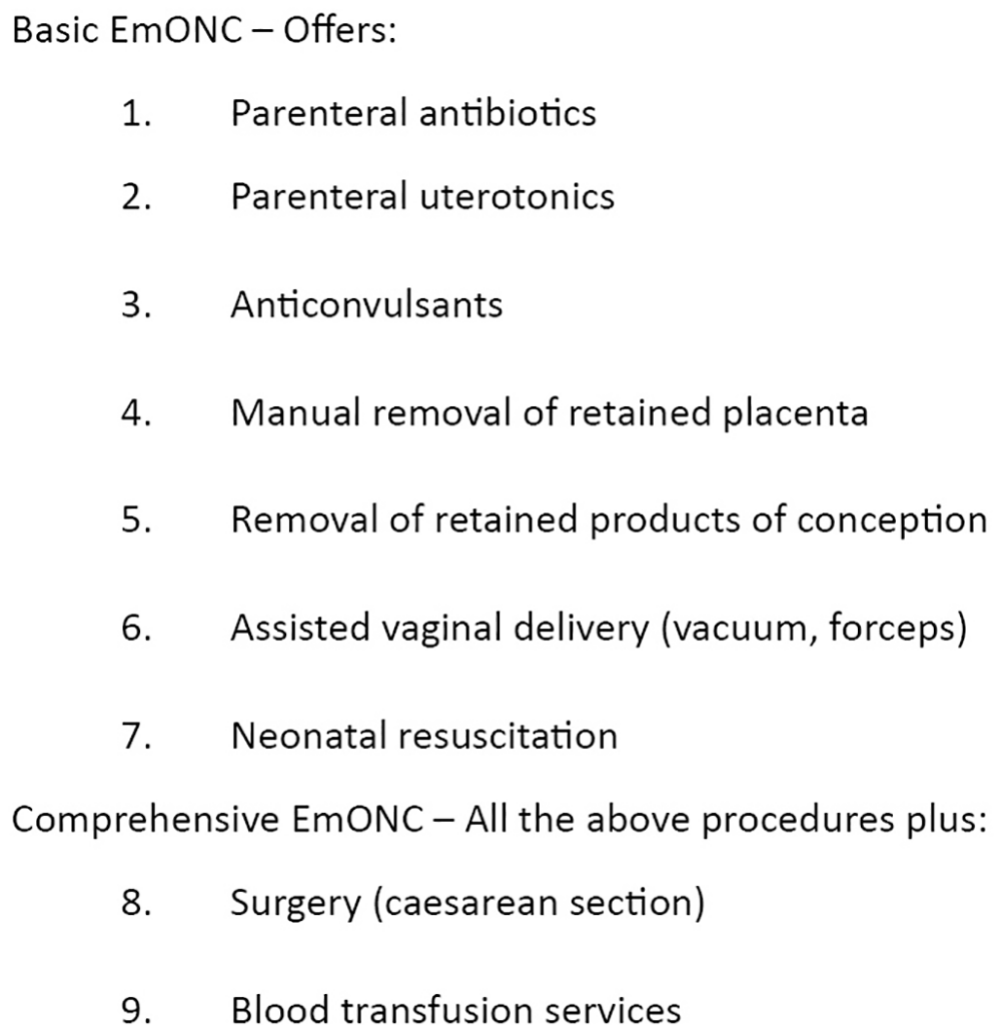
Basic and Comprehensive Emergency Obstetric and Neonatal Care.

EmONC refers to life-saving services for maternal and neonatal complications provided by a professional in a health facility as described in Fig 1 [7,8], and are essential in reducing maternal and neonatal mortality [2]. However, a large proportion of maternal deaths occur in hospitals compared to home [9]. This is related to delays in accessing skilled care as many arrive at a facility in critical condition or more women with complications are referred to hospitals, but also delays in receiving quality care after arriving at the health facility [9].

Monitoring short-term improvement in maternal health using maternal mortality ratio is challenging in low-income countries due to under-reporting, misclassification of maternal deaths, lack of resources to conduct large surveys, and census data are only available every ten years [10]. Output and process indicators have been proposed for monitoring interventions aimed at reducing maternal mortality [7,8]. One such indicator, the unmet obstetric need, quantifies to what extent emergency obstetric care is provided [11]. Thus, it measures the discrepancy between what should be done to deal with obstetric complications and the care actually provided [11].

The unmet obstetric need approach has been used to monitor responsiveness by the health system in emergency obstetric care in resource-limited areas [12,13]. Combining process indicators such as availability, accessibility and functionality of EmONC services, proportion of births in obstetric care facilities, met need for emergency obstetric care, caesarean section rate and direct obstetric case fatality rate is recommended to strengthen the assessment of maternal care services [14]. The Unmet Obstetric Need (UON) concept, developed by the UON Network [11,15], considers unmet need by including major obstetric interventions that are done for absolute maternal indications and the gap between those expected and those actually done. Process indicators do not focus on adequacy of bed capacity, competence and number of health care providers in health facilities. These become necessary as part of improvement to care when deficits are identified.

Zambia had an estimated maternal mortality ratio of 398 per 100,000 live births in 2014 [16]. Estimate from another source has not been included here as the DHS report so far presents estimates for comparison across countries. Although this may not be the most accurate considering the sisterhood method, challenges in reporting maternal deaths and large confidence intervals in the estimates, it is the most reliable for Zambia at present. Though 67% of women utilize health facilities at childbirth [16], urban and rural disparities exist. Our previous study in three districts of Kenya, Tanzania and Zambia revealed less health facility utilization at childbirth among women in rural than urban areas and in women of low socioeconomic status [17]. However, it is relevant to know the extent to which this low utilization encompasses women with life-threatening conditions and the difference in access to life-saving services between rural and urban areas. In Kenya, caesarean section rates were found to be much lower in rural than in urban settings [18]. In addition, poor attitudes towards provision of prompt attention and respectful care by health providers discourage women from seeking care at facilities [19]. Although underutilization of health facilities at childbirth in Zambia is known, less is known of the magnitude of unmet obstetric needs. We aimed to assess and compare the unmet need for life-saving obstetric services in rural and urban areas in a Zambian district by using the unmet obstetric need approach. Specifically, we aimed to obtain information on major obstetric interventions performed. Secondly, we aimed to assess the functionality of EmONC health facilities in rural and urban areas.

## Methods

### Study design

This was a retrospective cross-sectional facility-based study which was part of the ‘Response to Accountable priority setting for Trust in health systems’ (REACT) project. The project used an ethical framework of Accountability for Reasonableness (AFR) to guide achievement of a fair and legitimate priority setting process that would enhance trust, quality and equity in access to health care [20]. The project was designed to evaluate before and after effect of an intervention by implementation of the AFR process which has been described elsewhere [20]. This study analysed baseline data focused on emergency obstetric care. All facilities offering EmONC services in Kapiri Mposhi were enrolled in the study, that is, four rural health facilities and one urban hospital. The hospital was the main facility that offered childbirth services in the urban area. Two of the rural facilities were government run while two were faith-based.

### Study setting

The district Kapiri Mposhi, in the Central Province of Zambia, connects to the northern part of the country via road and railway network. The population was 240,000 in 2010, 35% being urban [21]. Urban was defined according to population census of 2000 as localities of 5,000 inhabitants or more and a majority of the labour force in non-agricultural activities [22]. Kapiri Mposhi is 17 219km2 with a population density of 14.7 persons per km^2^ [21]. The main economic activity is subsistence agriculture and small-scale trade in the urban area. Health services for childbirth are provided mainly by the public health sector in both urban and rural areas together with a small contribution by mission health facilities in the rural area [17]. The district of Kapiri Mposhi had no comprehensive EmONC service and was not providing caesarean sections at the time of the study. Thus, women with complications in pregnancy were referred and transported by ambulance to Kabwe General Hospital, 50km outside the district. Out of 27 rural and urban health facilities that offered childbirth services, only five offered EmONC services [23]. 37% women in rural and 77% in urban areas had health facility childbirth [17].

### The UON approach

The approach uses Major Obstetric Interventions (MOI) for Absolute Maternal Indications (AMI) to assess the health system’s ability to respond to complications in pregnancy and childbirth. Major obstetric interventions included caesarean section, laparotomy or hysterectomy for ruptured uterus, and destructive vaginal operation [11], i.e. life-saving commodities used for absolute maternal indications. This study included all these interventions except destructive vaginal delivery which was not a usual practise in the study setting. The assumption is that a minimum level of life-threatening complications in pregnancy and childbirth require major obstetric intervention. Studies have estimated that this minimum is 1.4% of expected births that require major obstetric interventions for absolute maternal indications in populations such as the study setting [24]. The unmet obstetric need is estimated as:

UON = 100% × (estimated expected numbers of MOI - actual numbers MOI carried out) / (estimated expected numbers of MOI).

The estimate could be viewed as an approximation since some limitations in the data are likely. The assumption is that the expected number of MOI for a given population over a period of time is known, and that the utilization of services for a particular problem, that is actual numbers of MOI, is also known. On one hand, the expected numbers of MOI is determined by the crude birth rate and the population size. Thus, an upward adjustment of these determinants would result in higher estimates of expected number of MOI and likewise a downward adjustment would result in lower estimates of expected number of MOI. On the other hand, the actual numbers of MOI which reflects the utilization of services for a particular problem depends on accurate recordings in registers and whether most women utilize the services within the area studied. If many of the recordings are inaccurate or inconsistent, or if many women with complications utilize services that are not part of the studied area, there could be an under-estimate of the actual numbers of MOI performed.

### Data collection

Data were collected in 2011. All recorded institutional deliveries from 1^st^ January to 31^st^ December 2010 in the EmONC rural health facilities and the urban hospital were obtained. Data on admissions, subsequent deliveries and referrals to the comprehensive EmONC service at Kabwe General Hospital were collected retrospectively and extracted by an obstetrician and two nurse midwives using a data abstraction form. Information on catchment area of origin, mode of delivery, obstetric complications, and outcome for both the mother and the newborn was also obtained. Sources of information were registers of maternity admission and delivery wards, and follow-up from maternity ward and operation theatre in the comprehensive EmONC facility at the general hospital. Further information was obtained from case records when register information was incomplete.

### Data Quality

**Fig 2:**
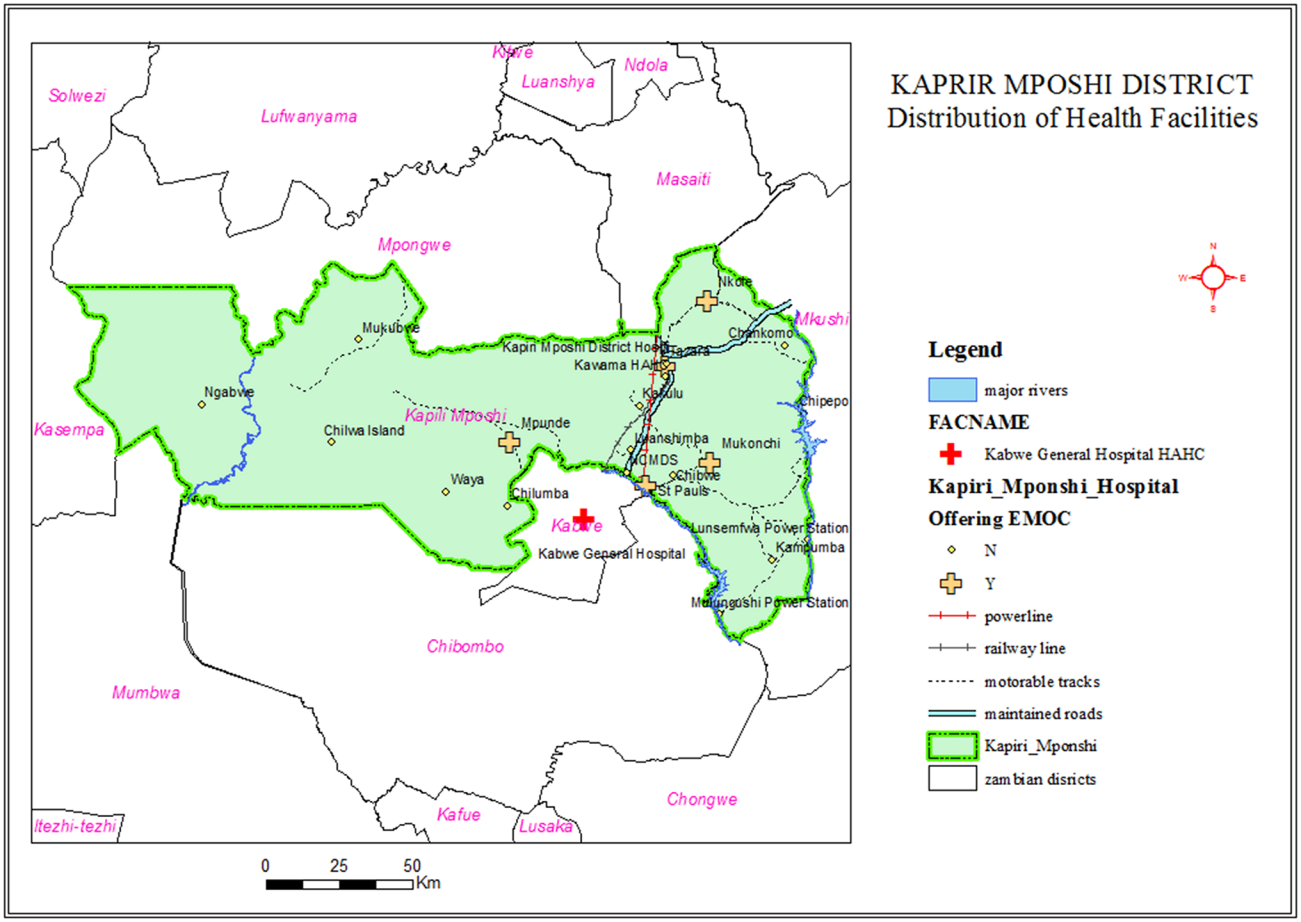
Distribution of health facilities with and without EmONC in Kapiri Mposhi, 2010.

Reliability of the data depends on reproducibility, which implies the ability of health care providers to notify the same complications in the same way. This includes whether the same health care provider (test-retest intra-rater reliability), and other health care providers (inter-rater concurrent reliability) notify complications in the same way. Validity of the data depended upon how reliable the recorded diagnoses were made, such as cephalo-pelvic disproportion which could have low specificity. Cephalo-pelvic disproportion is a common and accepted diagnosis and may be used by default in less clear conditions. However, a gauge of reliability and validity of data poses challenges when dealing with women who pass quickly through the system of care providers. On data completeness, it was possible that some women could have utilized other hospitals neighbouring Kapiri Mposhi district, although the geographical distribution of other hospitals made self-referrals less likely. Whereas Kabwe General Hospital was about 50km from Kapiri Mposhi, Ibenga Mission Hospital and Mpongwe Mission Hospital were 94km and 99km away, respectively, and public motor transport was not easily available for the local community (Fig 2).

Data obtained from registers mainly agreed with those recorded in case records, such that there was no reason to query the quality of information in the registers.

Information on functionality of basic and comprehensive EmONC facilities was also obtained. The rationale for this was based on knowledge that to reduce maternal deaths, certain obstetric functions in facilities must be available. Certain health facilities may be categorised as EmONC by the health system, yet if conditions are not met this may hide inadequacy in coverage of services. Fig 1 shows signal functions that describe basic and comprehensive EmONC facilities. A standard tool was used to obtain information on functions performed at least once in the previous three months prior to the survey by interviewing the nurse-in-charge of the maternity ward [8]. Reasons for not performing any of the signal functions were recorded. Inspection for availability of equipment and drugs in maternity wards was done. The WHO defines a basic EmONC facility as one that performed all seven signal functions in the last three months prior to a survey [8]. A comprehensive EmONC facility performs caesarean section and blood transfusion plus the seven functions in basic EmONC in the last three months. However, if a function such as assisted vaginal delivery is systematically absent in an area due to policy, the functioning of the facilities is indicated as “basic EmONC minus 1” or “comprehensive EmONC minus 1” [8].

### Data analysis

Data analysis was done using SPSS for Windows version 20, SPSS Inc. Chicago Illinois. Absolute maternal indications included uterine rupture; obstructed labour due to mal-presentation (transverse lie, oblique lie, shoulder presentation) and cephalo-pelvic disproportion; antepartum and severe postpartum haemorrhage; and abdominal pregnancy. For women who had more than one diagnosis, the main indication that led to intervention was used in the analysis. Maternal complications that could result in a vaginal delivery without causing a maternal death such as eclampsia and foetal indications such as foetal distress, breech presentation and cord presentation/prolapse were not included as absolute maternal indications. Major obstetric interventions included caesarean section, hysterectomy or repair for ruptured uterus and laparotomy for abdominal pregnancy. Abortions were excluded.

We compared age and parity between the rural and urban population by using independent t-test; origin of women, absolute maternal and foetal indications for interventions, and outcome of new-borns by using Pearson’s chi square test; and major obstetric interventions by using Fischer’s exact test. Origin of women meant residence either within the catchment area, from 12km to 35km radius of a health facility, or from outside the catchment area. Frequencies and proportions of complications were estimated for rural and urban health facilities. We also calculated the major obstetric interventions for absolute maternal indications as a percentage of population expected births (estimated by multiplying the crude birth rate with population size) [21], and calculated urban rural rate ratio. Based on the reference of 1.4% (95% CI 1.27% to 1.52%), the variance estimate for unmet obstetric need for the total study population was 40.8% (95% CI 34.9% to 45.8%). The variance estimate for the urban area was -48.6% (95% CI -66.7% to -37.5%), and the rural area was 72.6% (95% CI 69.8% to 74.8%). We assessed the functioning of the facilities as EmONC by finding out whether defining procedures were performed in the three months prior to the study.

### Ethical clearance

Ethical clearance was obtained from University of Zambia Research Ethics Committee. Permission was also granted by Kapiri Mposhi District Health Management office. This study was specifically approved by the Steering Committee of REACT project, which is also the project review board. Confidentiality and anonymity of study candidates was maintained.

## Results

### Characteristics of women with childbirth in EmONC facilities in urban and rural areas

A total of 2114 women in urban and 1226 in rural areas had childbirth in EmONC facilities (Table 1). Median age (inter-quartile range—IQR) was 24 (20, 30) years for both urban and rural areas. Primigravida constituted 32% and 25% of women in urban and rural areas respectively (Table 1). More women in rural areas had higher parity (four or more) compared to urban areas, 33% vs. 22% respectively. Seventy-one percent of women in the urban were from within the catchment area for the study facilities compared to 30% in the rural areas (Table 1). Caesarean section was performed on 3.4% of deliveries in both urban and rural areas (Table 1). Missing information on delivery mode was low, 1.8% in rural and 1.5% in urban areas, p = 0.534, rate ratio 1.19 (95% CI 0.69–2.06).

**Table 1:** Characteristics of women who had facility childbirths in EmONC facilities in Kapiri Mposhi district and referrals from Kapiri Mposhi to Kabwe general hospital, 2010.

	Urban hospital N = 2114 (%)	Rural health centres N = 1226 (%)	^†^p-value
Age (in years)*			
12–19	521 (24.8)	299 (24.7)	0.441
20–24	561 (26.7)	355 (29.3)	
25–29	495 (23.5)	218 (18.0)	
30+	528 (25.1)	340 (28.1)	
Parity*			
Primigravida	670 (32.3)	285 (25.0)	<0.001
1–3	942 (45.4)	481 (42.2)	
4+	464 (22.4)	375 (32.9)	
Origin of woman*			
Within catchment area	1497 (70.8)	369 (30.1)	<0.001
Outside catchment area	433 (20.5)	564 (46.0)	
From another district	34 (01.6)	188 (15.3)	
Not recorded	150 (07.1)	105 (08.6)	
**Normal deliveries**			
Normal deliveries	2008 (96.4)	1160 (96.4)	0.921
**Major Obstetric Interventions**			
Caesarean section	71 (3.4)	42 (3.4)	0.922
Hysterectomy/repair for ruptured uterus	2 (0.1)	1 (0.1)	
**Absolute Maternal and Foetal Indications**			
Absolute Maternal Indication (AMI)	86 (4.1)	34 (2.8)	0.275
Foetal indication^‡^	11 (0.5)	7 (0.6)	
Non-AMI (maternal indications)^§^	30 (1.4)	7 (0.6)	
Outcome of baby*			
Alive	1995 (96.8)	1166 (97.2)	0.592
Stillborn	59 (2.9)	32 (2.6)	

*Total numbers do not add up to n due to missing values

^‡^Foetal indications such as foetal distress, cord prolapse/presentation, breech presentation

^§^Non-AMI (maternal indications such as eclampsia)

^†^Pearson’s chi square test for independence was used for comparison of origin of woman, normal deliveries, absolute maternal and foetal indications, and outcome of baby; t-test was used for age and parity (continuous variables); Fischer’s exact test for major obstetric interventions due to values less than 5 in two cells.

### Functioning of EmONC facilities

All rural health facilities had administered uterotonic drugs (parenteral oxytocin) in the previous three months prior to the survey. All had performed manual removal of retained placenta and new-born resuscitation. Only one facility in the rural area had equipment and performed removal of retained products of conception. Parenteral antibiotics during pregnancy or puerperium had not been administered due to policy that recommended referral of women with sepsis, although they had administered to other patients with infection not related to pregnancy. Anticonvulsants for eclampsia were not administered due to lack of indication, i.e. no women with eclampsia/severe preeclampsia had presented to the facility in the three months prior to survey. Assisted vaginal delivery using vacuum extraction or forceps delivery was not performed due to policy that recommended referral for all women with prolonged labour. The urban hospital performed all functions except caesarean sections and women were referred by ambulance to the general hospital in the neighbouring district when there was need for a caesarean section. Thus, even though there were five facilities designated as EmONC per 240,000 population in 2010, the recommendation of at least one comprehensive and four basic EmONC facilities per 500,000 population was not met during the study period.

### Complications observed during childbirths in the facilities

**Fig 3:**
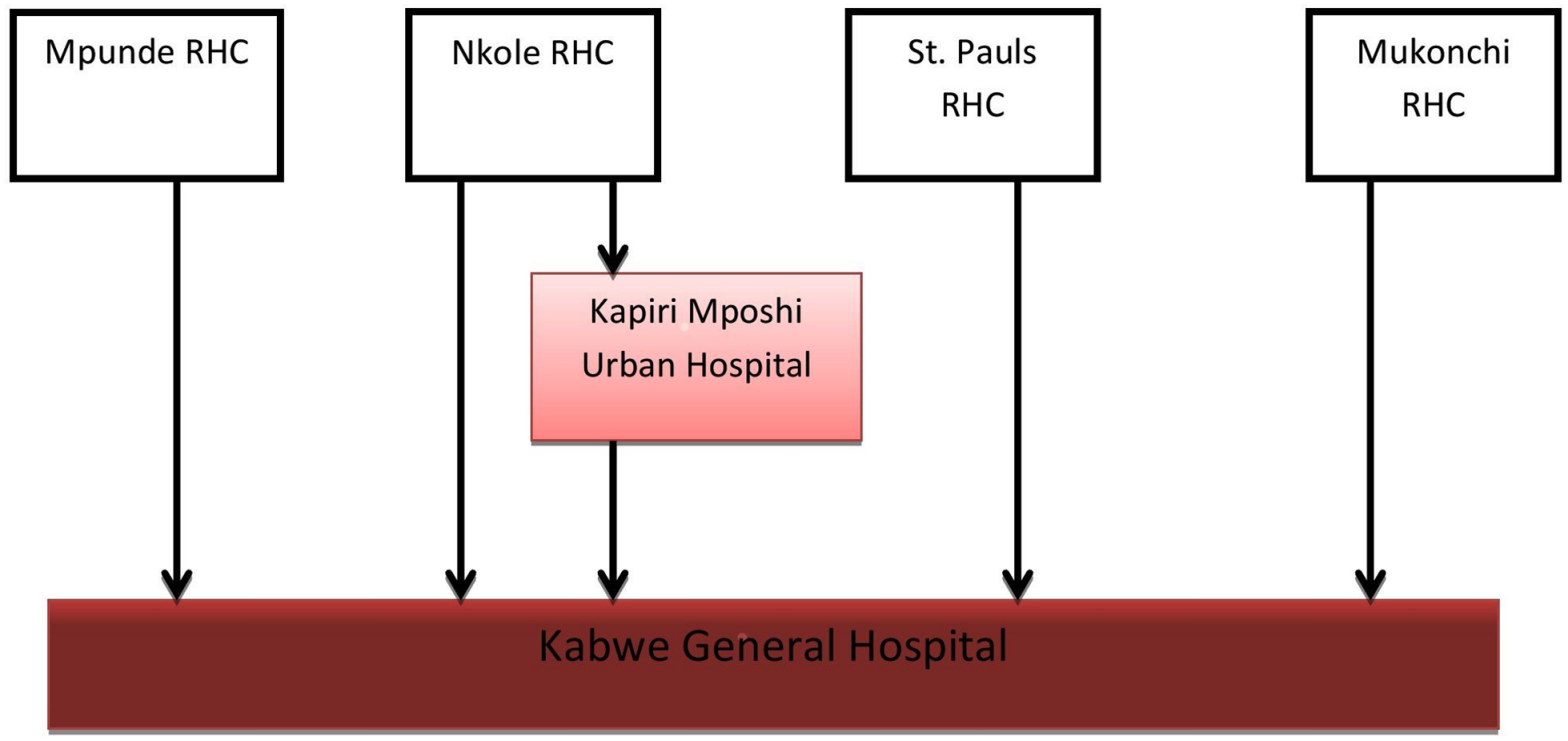
Flow of women with complicated institutional deliveries from basic to comprehensive EmONC facility in Kapiri Mposhi in 2010.

Cephalo-pelvic disproportion was the most common indication for a major obstetric intervention, 54% and 33% of all complications, in urban and rural areas respectively (Table 2). 3% of women presented with post-partum haemorrhage (Table 2). 7.1% of pregnant women from Kapiri Mposhi Urban Hospital were referred due to complications, whereas Mukonchi Rural Health Centre (RHC) referred 5.3%, Mpunde RHC 3.6%, Nkole RHC 2.6%, and 1.7% from St.Paul’s RHC. Women were referred directly to the comprehensive EmONC general hospital, except Nkole RHC that referred to the urban hospital due to proximity unless a major obstetric intervention necessitated referral to the general hospital (Fig 3).

**Table 2:** Frequency and proportions of complications in pregnant women who were referred to Kabwe general hospital from urban and rural EmONC facilities in Kapiri Mposhi, 2010.

Type of complication	Frequency	Total proportion (%) n = 175	Proportion in urban (%) n = 127	Proportion in rural (%) n = 48	Proportion of women with MOI* in urban (%) n = 71	Proportion of women with MOI* in rural (%) n = 42
Cephalo-pelvic disproportion	85	48.6	54.3	33.3	63.4	33.3
Antepartum haemorrhage	18	10.3	6.3	20.8	5.6	21.4
Postpartum haemorrhage	3	1.7	1.6	2.1	0.0	0.0
Mal-presentation^§^	11	6.3	3.9	12.5	5.6	11.9
Eclampsia	8	4.6	5.5	2.1	2.8	2.4
Uterine rupture	3	1.7	1.6	2.1	2.8	2.4
Foetal distress	8	4.6	5.5	2.1	5.6	2.4
Breech	7	4.0	1.6	10.4	0.0	11.9
Cord presentation/ prolapse	3	1.7	1.6	2.1	0.0	2.4

*MOI = major obstetric intervention (included caesarean section and repair/hysterectomy of ruptured uterus).

^§^Mal-presentation included shoulder presentation, transverse and oblique lie, and excluded breech.

Other referrals included women with high risk pregnancies such as previous caesarean section which constituted 14.1% and 11.9% in urban and rural areas respectively, of major obstetric intervention (MOI).

### Major obstetric interventions for absolute maternal indications

EmONC facility childbirths constituted 81% of expected births in urban and 16% in rural areas (Table 3). Based on the assumption that 1.4% of women would be in need of life-saving intervention [11], we calculated 142 women should have been referred for a major obstetric intervention, but only 84 had actually been treated. The overall unmet obstetric need in the population was 58 out of 142 women or 41% (95% CI 33–49%), in which the deficit from rural areas was estimated to be 77 out of 106 women or 73% (95% CI 71–75%). This implied that 77 women in the rural areas in 2010 in need of a life-saving intervention did not receive one and probably died or had severe morbidity. No deficit was observed in the urban area. The negative deficit implied that more major obstetric interventions for absolute maternal indications were actually performed than expected. This may reflect the possibility of some rural women providing a temporary address of residence that was close to the urban health facility. Major obstetric interventions for absolute maternal indications were 2.1% (95% CI 1.60–2.71%) of expected deliveries in the urban hospital, i.e. above the reference value of 1.4%, and 0.38% (95% CI 0.27–0.55%) in rural facilities (Table 3), implying unmet needs for women in rural areas. The rate ratio of major obstetric intervention for absolute maternal indication in urban to rural areas was 5.5 (95% CI 3.55–8.76).

**Table 3:** Major obstetric interventions (MOI) for absolute maternal indications (AMI) referred from urban hospital and rural EmONC centres in Kapiri Mposhi to Kabwe general hospital, 2010 (women aged 12–48 years).

Health centre/ hospital	Expected births in 2010^β^	Observed facility births in 2010	Need for MOI for AMI in 2010*	Actual MOI for AMI in 2010^§^	Number deficit^†^	Deficit (%)^‡^	MOI for AMI as a % of expected births^#^
Urban health facility	2617	2114	37	55	-18	-48.6	2.10 (1.60–2.71)
Rural health facilities	7557	1226	106	29	77	72.6	0.38 (0.27–0.55)
Total	10174	3340	142	84	58	40.8	0.83 (0.67–1.02)

*Need of major obstetric intervention for absolute maternal indication reference value taken as 1.4% according to the UON approach.

^β^Total expected births in the study population estimated by multiplying crude birth rate (42.6 per 1000) by population size (238826).

^§^Actual MOI for AMI include number of major procedures actually performed.

^†^Number deficit is the need for MOI in the study population less the actual MOI performed.

^‡^Deficit is the unmet need for major obstetric interventions in the study population calculated as number deficit divided by need for MOI and multiplied by 100 to give percentage. A negative deficit implies that more major obstetric interventions for absolute maternal indications were actually performed than the expected need.

^#^Actual major obstetric interventions performed for absolute maternal indications divided by expected births in the study population and multiplied by 100 to give percentage.

Absolute maternal indications (AMI) include women with a diagnosis that leads to maternal death if no operative intervention is performed. This excluded conditions such as eclampsia where women could be delivered vaginally without necessarily having caesarean section. In this study there were three maternal deaths arising from complications other than AMI, i.e. 1) pregnancy-induced hypertension, anaemia and congestive cardiac failure and was referred to a comprehensive EmONC, Kabwe general hospital, and delivered by caesarean section; 2) eclampsia and was referred to the comprehensive EmONC facility in Kabwe and delivered by caesarean section; 3) post-partum haemorrhage due to retained placenta after normal vaginal delivery at Kapiri Mposhi urban hospital.

## Discussion

Our study revealed a substantial deficit in the use of emergency obstetric services. However, it was the rural population that suffered the entire deficit, which was 73% implying that 77 out of 106 women had unmet needs in 2010. Further, the likelihood of undergoing a major obstetric intervention was 5.5 times higher in women from urban compared to rural areas, which underscores the unacceptably large inequity between urban and rural societies. In the rural area, only one health facility met the criteria of “basic EmONC minus 1”. The suffix “minus 1” was used due to absence of assisted delivery by vacuum or forceps in all rural facilities. The health policy did not recommend midwives to perform the procedure in health centres. Three rural health facilities did not qualify as “basic EmONC minus 1” since they lacked equipment to perform removal of retained products of conception.

Rural health facilities had few deliveries, 13–45 deliveries/month, and so could raise concern about skills and experience of the health providers [25], or trust from the community. Some facilities had not received and administered parenteral anticonvulsants for eclampsia in the three months prior to the study. This could reflect low utilization of health facilities for childbirth partly associated with constraints of long distance [17,26], and lack of trust due to perceived poor quality of care [19]. Our finding of 71% of women in the urban area residing within the catchment area compared to 30% in the rural area reflects that a higher proportion of women in the rural area probably have less geographical access to EmONC services than in urban areas. Furthermore, 16% of expected births in the rural and 81% in urban areas occurred in health facilities that were supposed to provide basic EmONC. Our previous study in the same area found 37% pregnant women in rural and 77% in urban areas had facility utilization during childbirth [17]. This could indicate that most institutional births in rural areas occurred in non-EmONC facilities, or possibly by-passed to urban facilities.

The possibility of by-passing rural facilities and providing a temporary urban address could not be ruled out, although most women who received a major obstetric intervention were traced within the health referral system. By-pass has been associated with perceived poor quality of care due to lack of emergency obstetric care services in other countries [27]. In Morocco, assessing unmet obstetric need at district level lead to improved quality of care and increased utilization of facilities [12]. Tanzania, Rwanda and Ethiopia also improved utilization of health facilities by women and achieved reduced mortality after upgrading infrastructure and technical competence of their health providers [28].

The substantial rural-urban disparity demonstrated in the present analysis of unmet obstetric needs should alert policy makers and the health care system to improve life-saving services to rural areas. The situation highlighted in this study could have changed due to opening of a district hospital in the study setting after our data collection. However, the extent of improvement especially in the rural area can only be ascertained by a follow-up study of the present situation. Although Kapiri Mposhi district embraced accountability and equity in priority setting, decisions were constrained by limited resources [29]. Sharing ambulance service among basic EmONC facilities located far apart was a challenge for prompt referrals. Using the unmet obstetric need approach, Mali identified problem areas and embarked on a nation-wide improvement in communication and transport, reduced financial barriers and was able to increase institutional deliveries and management of obstetric complications [3]. A greater reduction in maternal deaths was observed in women with haemorrhage, whose survival depended on prompt management and referral [3]. Urban and rural inequity could also be related to socioeconomic differentials, particularly in sub-Saharan Africa where extremely low caesarean section rates are observed among the very poor [30,31].

Population differences between rural and urban areas worsen the rural-urban inequity. Rural areas in Zambia have a higher percentage of women who begin childbearing between 15–19 years compared to urban areas, 36% vs. 20% [16]. This difference would contribute to a higher need for emergency obstetric care in rural areas since women aged 15–19 years are twice as likely to die from causes related to pregnancy and childbirth compared to women aged 25–29 years (Banda, R. et al., submitted), [32,33]. Although our data indicated a higher proportion of primigravida that utilized facilities in the urban than rural areas, this is not likely to reflect the actual differences due to the large urban-rural differential in facility childbirth and thus leading to selection bias.

The commonest indication for major obstetric intervention was cephalo-pelvic disproportion. This was consistent with findings in the region [18,34]. Furthermore, cephalo-pelvic disproportion may be used by default when other causes of prolonged labour have not been properly explored such as dystocia, mal-presentation or inadequate uterine contraction. The levels of caesarean section observed in the urban area were much lower than the suggested 5–15% reasonable to prevent both maternal and foetal complications [8]. Other countries such as China and Brazil are concerned with high caesarean section rates from increased provision of services and nearly universal hospital delivery [35] and demand from women [36]. It was unlikely that women in our context demanded caesarean section. Our study population culturally viewed difficult childbirth and operative delivery as related to marital infidelity and normal childbirth was a sign of strength [19]. In addition, facility childbirth was mostly in public facilities unlike private facilities which have been associated with high caesarean rates [31].

Despite cephalo-pelvic disproportion being the commonest complication, the most common direct cause of maternal mortality in sub-Saharan Africa is post-partum haemorrhage [5,37]. The finding of few women with life-threatening post-partum haemorrhage and ruptured uterus in our study may indicate either poor access to health services such that women died before reaching facilities, or they benefited from prevention of post-partum haemorrhage by active management of third stage of labour and timely referral. The deficits and rates of major obstetric intervention for absolute maternal indications observed in this study were similar to other UON studies in the region [13,38,39].

There were some methodological issues that merit mention regarding accuracy and completeness of facility records. Evaluating maternal health services by use of process indicators in order to make improvements depend very much on accurate records in health facility registers. The reliability may suffer when records are incomplete or duplicate, a danger when using several registers. In this study we attempted to avoid double counting by cross-checking names and file numbers recorded in both admission registers and delivery ward registers during data collection. Incomplete information in delivery registers was followed up by further checking case records. It was also important to have proper records of origin of residence to compare unmet needs between urban and rural areas [38]. Details for women who were referred to the comprehensive EmONC facility were obtained from both the basic EmONC and referral facility, which helped to identify residence of origin. When more than one indication for an intervention was recorded, the absolute maternal indication was utilized for the analysis.

A limitation of our study was the exclusion of abortions and ectopic pregnancies when assessing absolute maternal indications. Although abortions and ectopic pregnancies are important causes of maternal mortality, they may strictly speaking not be appropriate indicators of obstetric care. Abortions are also prone to inaccuracies in reporting. Complications may be over-reported from incorrectly reported incomplete abortions. In our study most women with abortions were managed at basic EmONC facilities and rarely presented with severe haemorrhage or sepsis. Therefore, excluding them would not have affected the estimates of absolute maternal indications requiring major obstetric interventions. Another limitation is that we did not consider quality of care in facilities, such as delays from admission or development of complications before referral to a comprehensive EmONC facility. Further, the diagnosis of cephalo-pelvic disproportion as an absolute maternal indication may be subject to misclassification. Some women may be referred for a ‘borderline’ cephalo-pelvic disproportion but turn out to have an uneventful delivery. This might bias the results towards a low ratio of major obstetric intervention for absolute maternal indication. Midwives diagnosed cephalo-pelvic disproportion by both clinical assessment and the use of a partogram as a tool for monitoring progress of labour, which is adequate.

Despite these limitations, the unmet obstetric need approach has been found to be useful in monitoring health system performance of maternal health services. This approach on its own may not identify which level in the system needs improvement. Therefore, combining assessment of services in facilities becomes important. In this respect, the approach to priority setting in the REACT study focusing on fairness and inclusiveness from different stakeholders may be useful [19]. The present results of rural-urban inequities in the district may be an eye-opener for both local and national authorities. Upgrading non-EmONC to basic EmONC facilities could improve services in rural areas and providing an ambulance to each basic EmONC health facility could ease referral and transfer of women with complications.

Although facility data are limited in generalizability due to population variation in use of services, the results of this study may be extrapolated to districts with similar context in access to and functionality of EmONC facilities.

## Conclusion

This study reveals a considerable unmet obstetric need in rural Zambia with a striking rural-urban inequity both in use of EmONC facilities and major obstetric interventions. The likelihood of having a life-saving intervention was 5.5 times higher for urban than rural women. This should alert health policy makers to improve access to life-saving services in rural areas.
